# Comparing the relationship between emotional responsiveness and psychopathy across assessment types: a systematic review and meta-analysis

**DOI:** 10.1017/S109285292500015X

**Published:** 2025-03-10

**Authors:** Hedwig Eisenbarth, Femi Carrington, Matthew S. Shane, Kasia Uzieblo, Sally Olderbak, David S. Kosson

**Affiliations:** 1 Victoria University of Wellington, Wellington, New Zealand; 2 Ontario Tech University, Toronto, Canada; 3 Vrije Universiteit Brussels, Brussels, Belgium; 4 Flemish Helpline 1712, Brussels, Belgium; 5 IFT Centre for Mental Health and Addiction Research, Munich, Germany; 6 Department of Psychiatry and Psychotherapy, University Hospital, Ludwig-Maximilians-University, Munich, Germany; 7 Rosalind Franklin University of Medicine and Science, North Chicago, USA

**Keywords:** psychopathic personality, measurement variance, affect, facets, questionnaire, emotion

## Abstract

Although psychopathic personality traits are widely reported to be related to reduced reactivity to emotion-eliciting situations, findings are not consistent. It has been argued that these differences could be related to variations in the way psychopathy is measured. To examine whether measurement variance resulting from the use of clinical assessment versus self-report assessment could be driving such differences, this systematic review and meta-analysis investigated the comparability of relations between psychopathic traits and responsiveness to emotion-inducing tasks for clinical versus self-report measures. The systematic review resulted in eight studies and 131 effect sizes, which included studies of emotion categorization, emotion regulation, decision-making, and executive functioning tasks. Robust Variance Estimation correlated effects models revealed no significant differences between effect sizes for clinical (PCL-R) versus self-report (PPI, SRP, and LSRP) assessment-based psychopathic traits and emotion tasks. Despite the small number of studies that included both clinical and self-report assessments of psychopathy, these results do not provide any evidence for an assessment-based difference in correlations with emotional responsiveness across tasks. The findings also show no associations between scores on emotional responsiveness and indices of psychopathy. Future research on emotional responsiveness in psychopathy should include both assessment types to be able to increase the research basis for the comparison.

## Introduction

The debate regarding the assessment of the psychopathic personality has been active since long before contemporary measures for assessing this personality syndrome were developed.^1^ The Psychopathy Checklist-Revised (PCL-R)^2^ is the most commonly used assessment tool in clinical practice and in research within clinical/forensic groups. Despite some criticism,^3^ PCL-R-based assessment of psychopathy has amassed considerable construct and criterion validity.^4^ ^–^^6^ However, in normative populations, the use of other assessment methods has become more prevalent, including other expert-rating measures,^7^ ^–^^9^ measures based on ratings by close relatives,^10^ measures based on ratings of interpersonal behavior^11^ and self-report measures.^12^

The use of self-report measures, in particular, has steadily increased over the past several decades. Researchers have argued that these self-report measures offer several advantages over the PCL-R, including less dependence on explicit criminal conduct, less dependence on collateral file information, and a less time-consuming assessment process.^12^ However, the extent to which scores obtained via self-report measures reflect the same underlying construct as the PCL-R remains an important concern.^13^ In particular, the ratings on the PCL-R appear to reflect two broad underlying dimensions of traits: Factor 1, the core personality traits; Factor 2, the antisocial lifestyle dimension. Some work has suggested that self-report indices of the core affective and interpersonal traits of psychopathy (Factor 1 of the PCL-R) share less than 3% of their variance with clinical/forensic indices of these traits.^14^ ^–^^16^ In addition, the two kinds of measures have demonstrated inconsistencies in the patterns of correlations with robust correlates of psychopathy, such as physiological reactivity, across different types of psychopathy measures,^17^ ^,^^18^ as well as several more peripheral correlates of psychopathy including risk-taking,^19^ ^,^^20^ behavioral inhibition^21^ ^–^^23^ and theory of mind.^24^ The reasons for these discrepancies remain a matter of debate. They may reflect: (a) true differences in the constructs measured by the two approaches, (b) natural variation associated with measuring the latent variable construct of psychopathy, (c) greater measurement error associated with one or the other measurement approach, or (d) differences between the characteristics of samples that have been studied and the larger populations of interest.

Differentiating between these potential alternatives is difficult because only studies with specific features can offer direct insights. In particular, to provide direct evidence, studies would need to have measured psychopathic traits via both assessment types and have evaluated the relationship between both indices of psychopathic traits and a third dependent variable (e.g., emotion processing). At this point, several such studies do exist; however, no systematic comparisons between the patterns of findings for these assessment types have been reported. To this end, we have undertaken a systematic review and meta-analysis of published studies to offer a preliminary evaluation of the consistency with which the PCL-R versus self-report measures of psychopathic traits are related to emotional reactivity. Our specific focus was on studies providing independent measures of emotion processing, based on the centrality of emotion processing in empirical research on, and in theoretical approaches to, psychopathy.^25^ ^–^^27^

### Clinical and self-report assessments of psychopathy

The first published clinical measure of psychopathy, based largely on Cleckley’s list of characteristics,^18^ was the Psychopathy Checklist (PCL).^28^ The PCL and its derivatives are considered clinical measures because they require substantial training to integrate information about trait dispositions across different domains of life (e.g., school history, work history, family relationships, romantic relationships, etc.) and across different sources of information (self-reports, behavioral observations, reports by others) and to assign ratings regarding a series of personality and behavioral dispositions. Standard use of these measures requires both an assessment interview and a review of institutional files.^29^

The PCL’s requirements of specialized training, in-depth interviews, and collateral information prompted researchers to develop new self-report measures of psychopathy to permit a less time-intensive assessment process that could facilitate the assessment of a wider range of individuals. Some of the most widely used self-report measures are the Psychopathic Personality Inventory-Revised (PPI-R),^30^ the Self-Report Psychopathy Scale (SRP-III,^31^ and its newer version SRP-4),^32^ the Triarchic Model of Psychopathy Model Questionnaire (TriPM),^33^ the Levenson Self-Report Psychopathy Questionnaire (LSRP/LPSP),^34^ and the Elemental Assessment of Psychopathy (EPA).^35^

Several criticisms of self-report measures of psychopathy have been raised. First, researchers have expressed concerns about the reliability and validity of self-reports due to the potential for response bias, false responses (lying), and responses that lack self-insight.^36^ ^,^^37^ Some of these concerns may be particularly relevant for individuals high in psychopathic traits, as such individuals may be more likely than others to present themselves strategically or to use deception and manipulation to persuade or conceal.^26^ Indeed, participants with psychopathic traits have demonstrated a superior ability to falsify their self-report scores when attempting to make a good impression.^14^ It is possible that such distortions are particularly salient in higher stakes situations (e.g., court versus research) and that psychopathy assessments for research and forensic practice could be managed to a certain degree through the use of response distortion scales that are embedded into some self-report measures.^38^ ^–^^40^ ^,^^41^

A second concern is the fact that each self-report measure conceptualizes psychopathic personality differently.^12^ For instance, the TriPM was conceptualized through consideration of common descriptions of psychopathy in the literature and neurophysiological markers.^33^ The LSRP was conceptualized to specifically distinguish between primary psychopathy and secondary psychopathy subtypes of psychopathy.^34^ However, newer analyses suggest a three-factor structure spanning Egocentricity, Callousness, and Antisocial factors. Most studies of psychopathy subtypes have suggested that offenders with both primary psychopathy profiles and secondary psychopathy profiles are high in both the interpersonal-affective and antisocial-lifestyle traits of psychopathy.^42^ ^,^^43^ ^–^^45^ The EPA was conceptualized to reflect the disorder as explained by the five-factor model of personality.^35^ Only the Self-Report Psychopathy Scale (in its many versions) was developed to match the four-factor model of the revised PCL (PCL-R).^2^

Perhaps not surprisingly, given these conceptual distinctions, the correlational overlap between total scores on the different self-report measures varies between 0.19 and 0.79.^46^ ^,^^47^ It appears likely that the low overlap between the assessment measures may contribute to the variability in the empirical correlates of psychopathy. To the degree the different measures of psychopathy are assessing different constructs, their associations with correlates of psychopathy may be expected to vary. At the same time, it could be argued that the variance between measures of psychopathic personality is also an advantage as it allows various conceptualizations of psychopathic personality to be described and tested.

Most importantly, consideration must be given to the degree of measurement variance between scores obtained through expert-rater measures, in which background information is aggregated by one or more trained evaluators based on interviews and files versus through self-report measures, in which an individual rates the extent to which specific statements apply to themselves. Given the differences in these assessment methods, one may not expect high correlations between the scores obtained through these different approaches. However, preliminary work in this area suggests moderate correlations between some self-report and PCL measures, including PCL total scores (0.29–0.44), and Factor 2 scores (0.38–0.68).^15^ ^,^^48^ ^–^^51^ It is in the assessment of Factor 1 traits that self-report scores correlate only modestly with clinical ratings.^14^ ^,^^15^ ^–^^17^ It is in the assessment of Factor 1 traits that self-report scores correlate only modestly with clinical ratings.^14^ ^,^^15^ ^,^^17^ Importantly, most of this work has been limited to correctional samples because completion of the PCL-R requires the availability of collateral information which is often difficult to obtain in community settings. Thus, a systematic investigation of the relationship between correlates of psychopathy as assessed with clinical versus self-report measures must include clinical samples.

### Emotion processing as a key feature of psychopathy

Low emotional reactivity is commonly considered to be a core component of the psychopathic personality that distinguishes individuals high in psychopathy from individuals with similar, but distinct, forms of impulsivity or behavior control problems.^52^ ^–^^54^ For instance, emotion processing differences seem to often differentiate those high in psychopathy from those with antisocial personality disorder without psychopathy (for a recent review, see^55^), particularly regarding physiological reactions to negative stimuli. As such, emotion processing differences form a key part of several etiological perspectives, including the general emotion deficit hypothesis,^26^ the integrated emotion systems model,^56^ and the fear deficit hypothesis,^27^ ^,^^56^ each of which emphasizes low physiological or behavioral responsivity to emotion-provoking cues in general, or to some kinds of emotion-provoking cues, is the foundation underlying psychopathic personality. Other contemporary theories, which emphasize different mechanisms, also suggest impairments in emotion processing under some conditions. For example, the affect regulation theory^57^ ^–^^59^ suggests automatic blocking and attenuation of emotional responses under specific circumstances. Similarly, the Impaired Integration Hypothesis^60^ posits information-processing impairments that reflect reduced connectivity within emotion-processing networks.^61^ ^–^^63^ Alternatively, the Motivational Framework for Psychopathy argues that individuals with psychopathic traits are chronically unmotivated, rather than entirely unable, to allocate processing resources towards emotional stimuli and do so freely when motivated or even instructed.^64^

However, despite the widespread emphasis on altered emotion processing in psychopathy, the empirical findings have been somewhat inconsistent. For instance, whereas some studies have reported reduced levels of self-reported fear in those high in psychopathy, systematic reviews and meta-analytic studies have found no differences in fear levels.^65^ ^,^^66^ Moreover, despite substantial evidence for behavioral, psychophysiological and neural anomalies related to deviant emotion processing, there have been substantial inconsistencies in findings across studies,^55^ ^,^^65^ including conflicting findings about emotion categorization,^67^ ^–^^69^ behavioral responses to emotional cues,^70^ ^,^^71^ and amygdala reactivity-related measures.^72^ ^–^^74^ This variability could have several sources: First, it could reflect variability among the tasks and stimuli employed in these studies. Indeed, investigations of emotional responding in psychopathy have included a wide variety of experimental paradigms, including spontaneous emotional reactivity to unconditioned or conditioned stimuli (e.g., to pictures, movie clips, or emotional facial expressions), implicit learning from emotional cues, the manifestation of empathy within complex social scenarios, and risky decision-making under certain and uncertain constraints. Moreover, dependent measures in these tasks have ranged from reaction times to categorization accuracies, to physiological reactivity, and to neural activity. A systematic analysis of findings should, therefore take task types and measurement designs into account,^75^ as they might refer to different aspects of emotion generation and regulation from an emotion constructivist point of view.^75^ ^,^^76^

A second potential source of variability is sample characteristics. Whereas a majority of studies to date have been conducted within forensic hospitals or incarcerated groups, a growing number of studies have investigated correlates of psychopathy within the general population, including undergraduate student groups. These groups may differ from forensic samples not only in antisocial behavior and socioeconomic status, but also in cognitive and emotion processing characteristics. Even within clinical samples, variability as a result of comorbid psychiatric disorders may contribute to diverse findings.^77^ ^,^^78^

Third, some discrepancies in the relationship between psychopathy and emotional reactivity might reflect variability in the types of assessment measures used. For instance, self-report measures of psychopathy that conceptualize the syndrome according to the Big Five traits (i.e., the EPA) may show relationships with emotional deficits that differ from those for self-report measures that conceptualize the disorder according to the PCL-R’s four-factor model (i.e., the SRP-III/SRP-4). Similarly, method variance between clinical and self-report measures of psychopathy may be expected to contribute to differences in convergent validity with measures of emotional function.

### Current study

It is possible to differentiate between these potential sources of variability through a systematic evaluation of studies that have collected both self-report and clinical methods of psychopathy. To this end, this systematic review and meta-analysis aimed to compare the magnitude of emotional responsiveness in psychopathy in studies that used clinical and self-report-based assessments, where we use the term emotional responsiveness to include both emotion processing performance and regulation success. However, we recognize psychopathy measurement selection could be related to sample selection because the PCL-R is primarily used in forensic samples where substantive collateral file information is generally more often available, whereas self-report studies are most often conducted within community or undergraduate samples. Seldom are both clinical and self-report measures of psychopathic traits employed within the same study.^79^ ^,^^80^ In addition, we included indices of the specific components of psychopathy (i.e., factors of the PCL-R) as a moderator in the analysis, using approximations of Factor 1- and Factor 2-related dimensional scores as calculated for the various self-report measures.

## Methods

### Literature search and study selection

The meta-analysis process closely followed the Preferred Reporting Items for Systematic Reviews and Meta-Analyses (PRISMA) statement guidelines^81^ and was not (pre-)registered. An electronic search of multiple databases (PsycINFO, Pubmed, and WebOfScience) was conducted to identify relevant journal articles. The search included terms related to the forensic assessment of psychopathy (PCL-R and CAPPS) and to emotion (emotion*, affect*, feeling*, fear*, anger*, sad* OR happ*, disgust*, surpris*, contempt*, shame*, guilt*, empath*, valence, pleasure*, displeasure*, arousal*, amusement, contempt, pain*, anxiety, and anxious) that were published in English before March 16, 2023.

### Inclusion and exclusion criteria

Studies were required to meet the following inclusion criteria:

- a psychopathy assessment via a clinical tool (e.g., Psychopathy Checklist-Revised) and potentially reporting additional self-report measure data (e.g., Psychopathic Personality Inventory-Revised);
- behavioral or physiological data intended to evaluate some facet of state emotion processing; and
- sample is primarily adult participants (mean sample age 18 years).

Studies were excluded if they had the following characteristics:

- dissertations, preprints, or unpublished studies that had not undergone outside peer-review;
- use of the PCL-YV to assess psychopathic traits;
- measures of emotion processing were based solely on self- or other-reports or on measures of traits (e.g., parent or peer ratings); and
- neuroimaging, psychophysiological, or genetic data were excluded in the final stages of the screening process due to the limited number of studies reporting these kinds of data (e.g., neuroimaging (*k* = 2), psychophysiological (*k* = 1)). For cases in which a study reported these data types *and* behavioral data, the behavioral data were included.

### Article screening process

**Figure 1.**
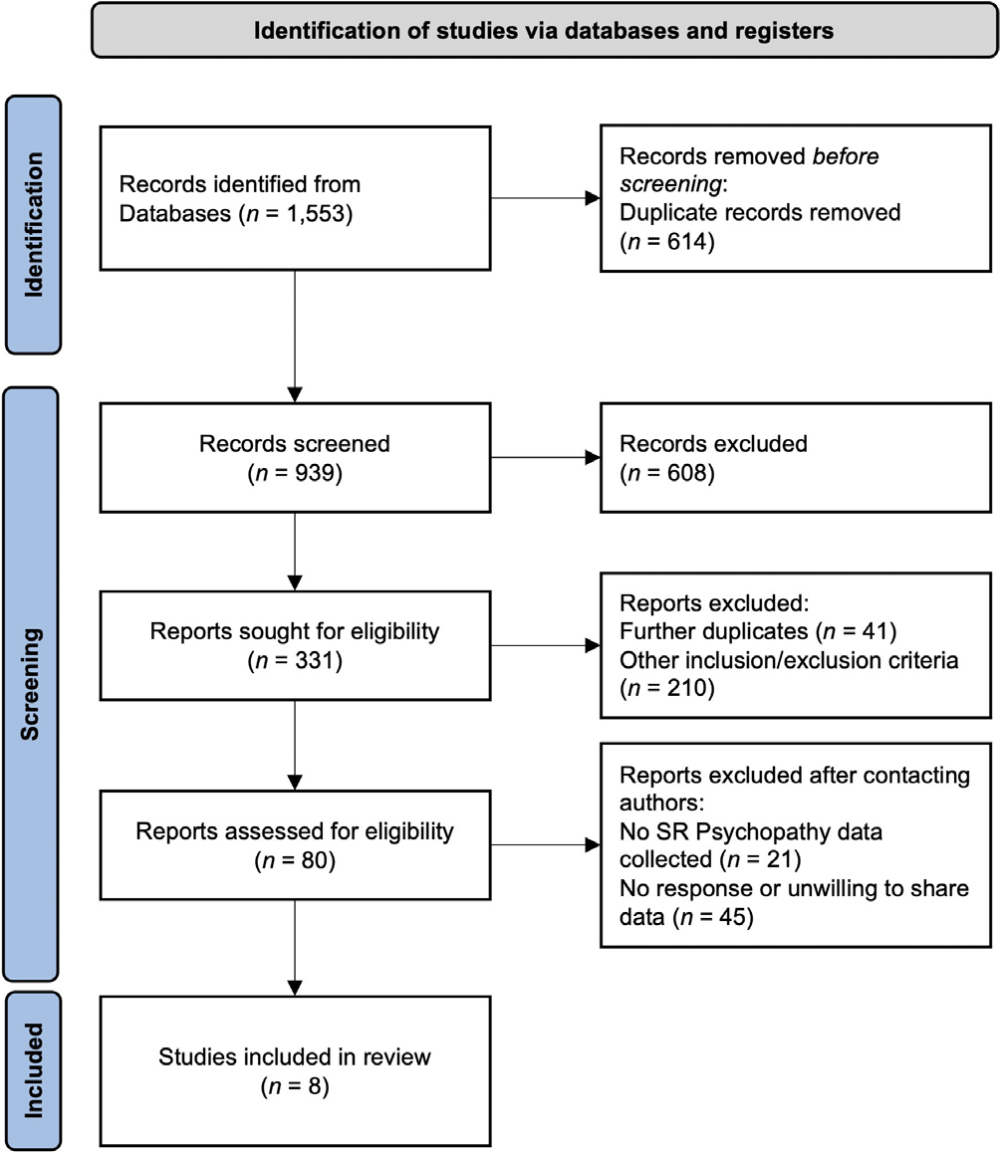
PRISMA chart for the screening process.

The initial database search elicited a total of 1553 articles. Each of these articles was screened by at least two independent raters via the following two-phase process. In the first abstract/title search phase, two raters independently reviewed each article based on the contents of their titles and abstracts. At this phase, articles were screened to ensure broad alignment with the *inclusion criteria* of the meta-analysis (see above). Articles were removed if they were duplicates (*n* = 614) or did not collect data from a task intended to assess a facet of emotion processing (*n* = 608). Following this initial screening process, 331 articles were retained for full-text review. During this second phase, the full text of each article was independently rated by two pairs of raters (i.e., H.E./M.S. or D.K./K.U.), and articles were assessed to ensure alignment with study inclusion and exclusion criteria. Inter-rater reliability between the two raters was generally high, with Cohen’s Kappa = 0.83. When the two reviewers disagreed, a third member of the team served as the final arbiter regarding the inclusion/exclusion of that article. Through this process, an additional 251 papers were excluded (41 were duplicates not identified during first-phase screening; 210 did not pass at least one inclusion/exclusion criterion; see PRISMA chart in Fig. 1).

This process left 80 articles for potential inclusion in the meta-analysis. However, of these 80 studies, only two articles^49^ ^,^^50^ explicitly reported data from *both* clinical and self-report psychopathy assessments; the other 78 studies reported clinical data and did not mention whether a self-report measure had been included. In case some studies that reported clinical data had also collected self-report data that were not reported in the final published manuscript, we contacted the corresponding author of each of these 78 papers to ask whether self-report assessments had been collected. This process led to the inclusion of six additional papers. (Two of these papers^29^ ^,^^82^ contained overlapping samples and were coded as the same study for analyses.) Thus, eight studies were included in the final meta-analysis (see Figure 1).

### Coding/scoring

#### Psychopathy assessments

All included studies assessed psychopathy via a variant of the Psychopathy Checklist (i.e., PCL-R, PCL:SV). Included studies made use of one of three different self-report psychopathy instruments, the Psychopathic Personality Inventory (PPI or PPI-R), the Self-Report Psychopathy Scale (SRP-III), or the Levenson Self-Report Psychopathy Questionnaire (LSRP; see Table 1). Comparisons between these forensic/self-report instruments are complicated by the fact that the PCL-R yields not only total psychopathy scores but also Factor 1 and Factor 2 scores (the broad dimensions of Factor 1 and Factor 2 in the PCL measures have been subdivided into four facets, which are not addressed in this meta-analysis), whereas some self-report instruments (e.g., PPI-R; LSRP) favor a different structure. To aid comparisons, we aligned the indices for all measures with the two-factor structure of the PCL-R. For the PPI-R, we coded combined scores on the Fearless-Dominance and Coldheartedness subscales as an index for “Factor 1” traits and accepted the mean Self-Centered Impulsivity subscale as an index for “Factor 2” traits.^14^ Similarly, the original LSRP primary and secondary factors^34^ were coded as “Factor 1” and “Factor 2” traits respectively.^83^ The SRP affective and interpersonal factors were aggregated to provide a measure of Factor 1 traits, and the lifestyle and antisocial factors were similarly aggregated to provide a measure of Factor 2 traits.^14^

**Table 1.** Demographic information and sample characteristics of included articles

Study	N	k	Sample type	Mean age	Gender (% M)	Task	Dependent Variable	Self-report measure	PCL version
Veit et al.^84^	14	21	Offending	43	100	Fear conditioning	Conditioned stimulus valence rating	PPI-R	PCL-R
Dargis et al.^85^	58	6	Offending	32	100	Facial emotion recognition	Fixations in mouth and eye regions	PPI	PCL-R
Sandvik et al.^24^	80–84	12	Offending	34	100	Reading the Mind in the Eyes	Accuracy	SRP-III	PCL-R
Book et al.^86^	57–59	18	Offending	33	100	Emotion identification	Accuracy for intensity ratings	LSRP	PCL-R
Psederska et al.^87^	672	6	Community	*	*	Iowa Gambling	Advantageous - disadvantageous choices	LSRP	PCL-SV
	645	6	Community	*	*	Cambridge Gambling	Tendency to bet on the more likely outcome	LSRP	PCL-SV
	645	6	Community	*	*	Cambridge Gambling	Average points after selection of most probable result	LSRP	PCL-SV
	693	6	Community	29*	64*	Balloon Analog Risk	Adjusted number of pumps on unexploded balloons	LSRP	PCL-SV
	680	6	Community	*	*	Go/NoGo	False alarms	LSRP	PCL-SV
Kennealy et al.^46^	430	7	Offending	*	100	Lexical decision-making	Accuracy	PPI	PCL-R
	1128	7	Offending	30*	100	Go/NoGo	False alarms/punished responses	PPI	PCL-R
Shane and Groat^29^	67	18	Offending	33	84	Emotion regulation	Subjective emotion ratings	LSRP	PCL-R
Denomme et al.^82^	105	12	Offending	36	67	Cue-elicited craving	Valence ratings	LSRP	PCL-R

*Note*: * demographics only available for full sample, not for subsamples; PPI = Psychopathic Personality Inventory, PPI-R = Psychopathic Personality Inventory-Revised, LSRP = Levenson Self-reported Psychopathy Scale.

#### Emotion measures

All eight studies retained in the meta-analysis included a task that assessed an aspect of emotion processing via behavioral or physiological metrics. The studies fell into three broad categories of emotion processing. Several of the studies presented participants with performance-based tasks in which they had to process emotionally charged information. Several other studies examined participants’ responsiveness to a stimulus or instruction designed to increase a kind of emotional response. More concretely, three reported data from emotion identification tasks, two reported data from behavioral impulsivity tasks (e.g., Go/No-Go task, Iowa Gambling Task), two reported data from emotion regulation tasks, and one reported data from a fear-conditioning task (see Table 1 for a detailed task description). For each task, we chose to include the most central indices of emotion processing within the meta-analysis. We coded effects so that positive effects would indicate relationships between greater levels of psychopathic traits and better emotion processing or greater responsiveness, and negative effects would indicate relationships between greater levels of psychopathic traits and poor emotion processing/less responsiveness. For emotion identification tasks, we included performance (e.g., accuracy, reaction time) on positive emotion, negative emotion, and neutral trials. For Go/No-Go task performance, we included the number of false alarms/commission errors and converted the effect sizes so that negative effect sizes would indicate that lower performance (more false alarms) was related to higher psychopathy scores, whereas positive effect sizes would indicate that better performance (fewer false alarms) was associated with higher psychopathy scores. For Iowa Gambling Task performance, we included net scores, subtracting the number of disadvantageous choices from the number of advantageous choices. For Cambridge Gambling Task performance, we included the tendency to bet on the more likely outcome and the average points after selection of the most probable result. For Balloon Analog Risk Task performance, we included the adjusted number of pumps on unexploded balloons and converted the effect sizes so that negative effect sizes would indicate more risky behavior (more pumps) being related to lower psychopathy scores. For lexical decision-making tasks, we included accuracy. For emotion regulation tasks, we included emotional state ratings on trials in which participants were asked to increase or decrease their emotional response to emotional pictures or just observe pictures (neutral). For craving-eliciting tasks, we included subjective stimulus ratings. For fear conditioning, we included ratings for the conditioned stimuli for both early and late acquisition phases (see Table 1).

We intended to subdivide the studies further based on the specific type of emotional task used. However, the small number of included studies precluded further subcategorization. Thus, the effect sizes from each of the eight studies were assigned equal weight within a single-parent analysis (see Meta-Analytic Effect Size Calculations below). In addition, we ran the meta-analyses based only on clear emotional responding tasks by excluding Go/NoGo and risk tasks from a supplementary analysis (Supplementary Materials).

### Data extraction

For each study, we extracted correlation coefficients (*r*s) between psychopathic traits (total and factor scores) and each metric of emotional responsiveness, with positive correlations indicating higher emotion categorization accuracy or higher intensity ratings being related to higher psychopathic trait scores. This resulted in several effect sizes per study. In addition, we extracted demographic information (e.g., mean age and gender composition) and sample characteristics (e.g., sample size and population type) from each article.

### Meta-analytic Effect Size Calculations

The meta-analyses were conducted in RStudio^88^ using Robust Variance Estimation (RVE) correlated effects models with small sample corrections in the *robumeta* package (version 2.0).^89^ RVE is a technique that considers the correlation within studies when estimating the variance of meta-analytic effect sizes by assuming a constant within-study correlation among the effect sizes.^14^ This makes the analyses robust to the violation of independence assumption of traditional meta-analytic techniques and allows the inclusion of multiple effects from a single study.^90^ ^,^^91^

The following steps comprised our analytic pipeline. First, we conducted a meta-regression to identify pooled effect size estimates to assess the overall relationship between psychopathic traits and emotional responsiveness. Second, we conducted separate meta-regressions to estimate the size of these relationships associated with clinical versus self-report measures of psychopathic traits. Third, we added an assessment-type moderator to examine the robustness of any differences in the relationship with emotional responsiveness for clinical versus self-report measures of psychopathy.

## Results

### Descriptive Statistics

The systematic review resulted in eight studies, yielding 131 effect sizes. The studies included predominantly participants from male offending populations (see Table 1) and had a mean sample size of 274.84 (*SD* = 328.58, range: 14–1,128). The mean age of participants across these included studies was *M* = 33.60 years (*SD* = 4.73, range: 28.57–43.14). The self-report measures involved in the eight studies included the PPI, the PPI-R, the SRP-III, and the LSRP.

We assessed for potential outliers using boxplots and normal Q–Q plots from the *stats* package in R (version 2.1.9^92^). No outliers were detected (see Supplementary Figures S1 and S2), and all effect sizes were included.

### Meta-analyses

#### Models assessing total psychopathy scores

**Figure 2.**
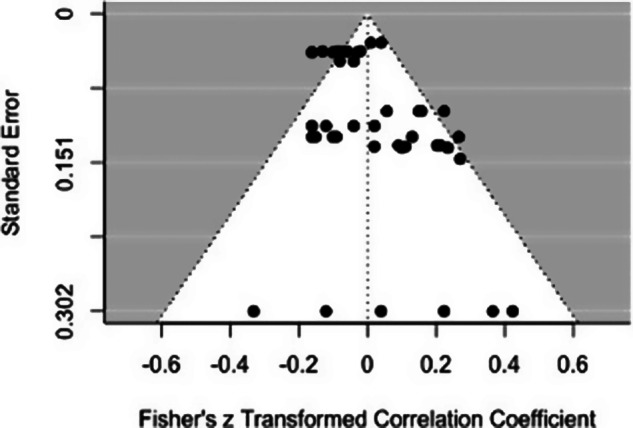
Funnel plot for total score effect sizes

The level of heterogeneity was large, with approximately 49.48% of variation in effect size estimation attributable to variation among studies.^93^ The funnel plot also shows moderate indications of publication bias (Figure 2). The overall relationship between total psychopathy scores and emotional responsiveness was not significant (*k* = 42, pooled *r* = −0.01, *p* = 0.79, 95% CI[−0.11,0.09], *I^2^* = 49.48). This was true of the pooled relationship across both assessment types (pooled *r* = −0.03, *p =* 0.08, 95% CI [−0.11, 0.05]), and also when psychopathy was assessed via clinical measures (pooled *r* = −0.03, *p* = 0.49, 95% CI[−0.13, 0.07], *I^2^* = 44.03) or via self-report measures (pooled *r* = 0.03, *p* = 0.66, 95% CI[−0.13,0.18], *I^2^* = 60.08). The heterogeneity in these separate analyses remained large. There was no statistical difference depending on the type of psychopathy assessment (*t*[4] = 1.21, *p* = 0.30, CI[−0.06,0.15]).

#### Models assessing factor scores

**Figure 3.**
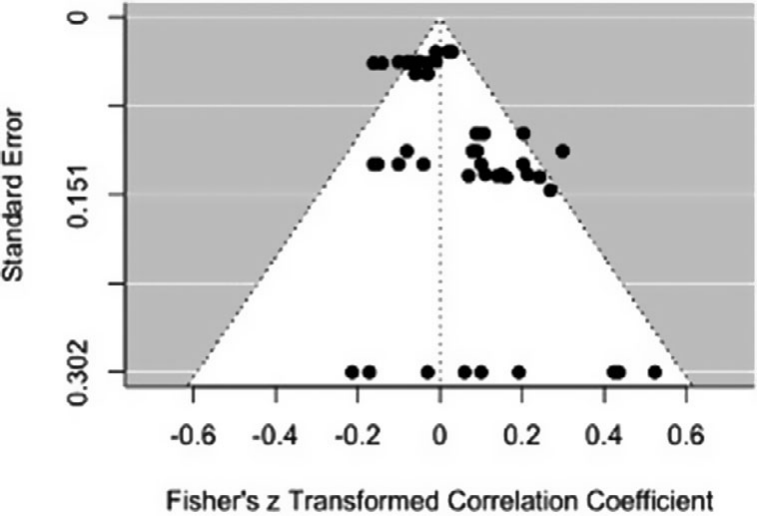
Funnel plot for Factor 1 effect sizes

**Factor 1.** The level of heterogeneity was moderate, with 42% of variation in effect size estimation attributable to variation among studies.^93^ The funnel plot (Figure 3) indicated little evidence of publication bias. Overall, we identified no significant relationship between Factor 1 scores and scores on emotional responsiveness (*k* = 48, pooled *r* = 0.02, *p* = 0.66, 95% CI [−0.09, 0.13]). Similarly, when broken down by assessment type, there was no reliable relationship between Factor 1 scores and emotional responsiveness when assessed via clinical measures (pooled *r* = 0.002, *p* = 0.96, 95% CI [−0.11, 0.11], *I^2^* = 37.95) or when assessed via self-report measures (pooled *r* = 0.02, *p* = 0.70, 95% CI [−0.12, 0.16], *I^2^* = 55.47). The magnitude of the relationship did not differ significantly as a function of the psychopathy assessment type (*t*(4) = −0.42, *p* = 0.70, CI [−0.09,0.06]).

**Figure 4.**
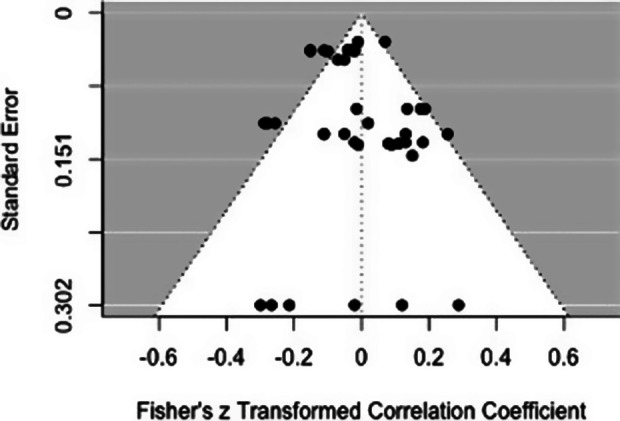
Funnel plot for Factor 2 effect sizes

**Factor 2.** The level of heterogeneity was moderate, with 61% of variation in effect size estimation attributable to variation among studies.^93^ The funnel plot (Figure 4) indicated little evidence of publication bias. We found no evidence of a relationship between Factor 2 scores and emotional responsiveness (*k* = 41, pooled *r* = −0.03, *p* = 0.43, 95% CI[−0.12, 0.06]). This was also the case when the effects of psychopathic traits were compared separately for clinically based (pooled *r* = − 0.04, *p* = 0.39, 95% CI [−0.14, 0.07], *I^2^* = 60.78) or self-report-based (pooled *r* = −0.02, *p* = 0.54, 95% CI [−0.12, 0.08], *I^2^* = 16.62) measures. The magnitude of the relationship did not differ significantly as a function of the psychopathy assessment type (*t*(4) = 1.28*, p* = 0.27, CI [−0.08,0.21]).

For additional analyses excluding effect sizes from gambling tasks, Balloon Analog Risk Tasks, and Go/NoGo tasks, see Supplementary Materials.

## Discussion

This study sought to evaluate the extent to which self-report measures of psychopathic traits and clinical measures of psychopathic traits would reveal comparable associations with indices of emotional responsiveness on laboratory tasks. The current systematic literature review yielded eight studies that collected both self-report and clinically based measures of psychopathy, among which the magnitude of relationships between psychopathy, using each type of assessment, and emotional task performance was compared. The analyses indicated that the prediction of emotional task performance was similar across both types of overall psychopathy assessment measures. Similarly, the effect sizes were not significantly different for either measures of the affective interpersonal core of psychopathy or of the antisocial lifestyle features of psychopathy. In addition, the present analyses indicated that the association between psychopathy and emotional responsiveness across the two assessment methods was not significant.

### Relationships between Specific Dimensions of Psychopathy and Emotional Reactivity

Analyses failed to provide evidence that either overall levels of psychopathic traits, or levels of Factor 1 or Factor 2 traits, were associated with differences in emotional responsiveness across methods. This finding of no differences across measures is surprising given the differences previously outlined between self-report and clinical measures.^65^ ^,^^94^ Differences associated with personality assessment methods may add unwanted variance,^13^ which may also tend to reduce the similarity of relationships for studies using these two kinds of measures. Moreover, there are only relatively small to medium correlations between scores based on clinically based versus self-report- and other report-based assessments of psychopathy.^95^ Thus, the present meta-analytic results, which found consistently no significant association between emotional task performance and psychopathy across both assessment methods, should not be affected by such bias. One possible explanation is that the relationship between psychopathy and emotional responsiveness may be less robust than is generally assumed,^72^ especially given that the non-significant effect of psychopathy may be partly related to a large variation in effect sizes, ranging from moderately positive to moderately negative values (see funnel plots in Figures 2–4). This pattern suggests the possibility that the relationship between emotion reactivity and psychopathy may vary not only in strength but also in direction. Clearly, it would be unwarranted to interpret the results of the current meta-analyses as providing a firm answer to the question of the relationship between psychopathy and emotion. There are too few studies to permit analyses of several dimensions of emotional responsiveness that appear fundamental, including the distinction between emotional impairment on performance measures and various indices of responsiveness to external and internal stimuli. The evidence linking psychopathy to emotional impairments is dramatic and substantial. At the same time, we acknowledge that several recent reviews have concluded that the relationship between psychopathic traits and emotion processing impairments is less robust than sometimes assumed.^65^

Alternatively, it may be that the results from the eight studies included in this meta-analysis are not representative of the broader literature on emotional differences in psychopathy. Indeed, several studies using only one type of assessment have at times identified different patterns of findings when using clinical measures versus self-report measures of psychopathy.^96^ ^–^^99^ These reports suggest the likelihood that systematic comparisons may well yield evidence of differences in the relationships of psychopathic traits with some kinds of dependent variables. Even so, based on the current evaluation of eight studies, the differences in the relationships for these clinical and self-report and clinical measures were generally quite small as well as not reliable in this meta-analysis.

In addition, the factor-level analyses suggested no association between the level of interpersonal affective traits and indices of emotional reactivity or between the level of antisocial lifestyle traits and emotional reactivity. These findings are also somewhat unexpected given the frequent reports that both these dimensions have been commonly linked, in opposite ways, to emotional reactivity. It is particularly the Factor 1 traits that are said to be markers of attenuated affective reactivity.^51^ However, Factor 2 traits have been somewhat consistently linked to heightened affective reactivity; in contrast to Factor 1, this relationship is typically positive.^100^ ^,^^101^ However, our meta-analytic findings do not indicate a relationship of emotional reactivity with either of the two factors.

Only a small number of studies have included (and reported) information about both clinical and nonclinical measures of psychopathy and emotion processing. In short, despite the large number of published studies on this topic, and despite the centrality of this relation in leading theories, few studies have explicitly considered the comparability of the nomological network generated by research using these different kinds of measures.^102^ ^,^^103^ It remains critical to examine the comparability of different kinds of psychopathy measures across other domains in which psychopathy has been argued to be reliably associated with specific psychological and physiological outcomes. If future studies continue to provide evidence of no differences in the size of relationships between psychopathy and scores on other components of the nomological network surrounding this syndrome, then these studies will suggest the possibility that findings obtained from studies using self-reported versus clinically based measures may be comparable. If those two assessment approaches may be considered to provide equivalent measures of interpersonal and emotional differences in psychopathy, researchers might want to avoid redundancy in data collection situations that place substantial demands on participants. It might also contribute to the choice of measure depending on the sample type, where some individuals might struggle with completing written questionnaires. However, as a recent meta-analysis points out, the relationships between scores on self-report and interview-based measures of psychopathy are relatively modest and vary depending on the PCL measurement type.^95^ Therefore, researchers are likely to design future studies to include both measurement types - regardless of the results from our meta-analysis - to increase the pool of studies on which conclusions can be based. In addition, due to the small number of studies and the limited variation of self-report measures involved, we were constrained to a focus on only the broad dimensions of Factor 1- and Factor 2-related effect sizes. Future studies based on a larger number of effect sizes could run self-report specific meta analyses.

As indicated by the substantial heterogeneity of the effect sizes we examined, the eight studies varied considerably with respect to the way emotional response was examined. In four of the studies, the dependent variables reflected performance with higher scores indicating superior performance (e.g., higher accuracy, faster response latency). In the other four, the dependent variable was more directly related to reactivity to an emotional trigger (including aversive conditioning, risky behavior, and reported difficulty regulating emotion). In addition, one study employed an instructed effort to increase or decrease emotional reaction to a stimulus. Although it could be argued that implicit emotion regulation is pursued at all times,^104^ contexts providing instructed emotion regulation are likely to involve a different processes than implicit emotion contexts.^105^ ^,^^106^ In fact, a recent meta-analysis identified a significant pooled impairment in emotion dysregulation related to psychopathic traits.^107^ However, the number of studies associated with each subgroup was not sufficient to conduct subgroup analyses, and therefore, future studies should more often include both types of assessment to allow meta-analytic studies to address differences between self-report and clinical measures of psychopathy with more narrow categories for emotional responsiveness versus adequacy of emotion-based information-processing.

### Limitations

This meta-analysis has several limitations. The goal of comparing relationships for clinical and nonclinical measures of psychopathy in the same sample led to only a small set of studies meeting inclusion criteria and to insufficient power to address differences in task types through moderation analyses. Although our analyses of heterogeneity do not suggest a strong publication bias, the effect sizes included in the meta-analyses are based on diverse dependent variables, including reaction times, accuracy, and subjective emotion ratings across various types of behavioral tasks. In addition, planned analyses that would test for differences between task types were not possible. In addition, it appears likely that there exist additional data that could not be obtained from the authors. As we acknowledged in our introduction, our factor-specific analyses were limited by differences in the approaches taken to construct self-report measures of the dimensions of psychopathy. In particular, whereas some measures were designed based around clinical (i.e., PCL) measures of psychopathy (e.g., the SRP), others were based on other perspectives (e.g., the Big-Five theory) or atheoretical clinical descriptions (e.g., the PPI). The extent to which the different measures are assessing only partly overlapping constructs limits the ability of any analysis to identify systematic differences across these kinds of measures. Another limitation is that the decision to only include published studies might have restricted the availability of data for this study. However, given the inclusion criteria, one important result from this systematic review and meta-analysis is the scarcity of emotion-processing studies that have included both clinical and self-report measures of psychopathy.

### Conclusions and future directions

The empirical literature on psychopathy and emotion processing has been largely divided into studies that employ clinically based assessments for psychopathy within clinical populations and employ self-report assessments within normative populations. The extent to which the findings across those two overlapping but distinct bodies of literature can be compared was unclear. The current findings provide important new evidence that across a subset of research – the extant studies that have included both clinically assessed and self-reported psychopathic traits – the relationship between psychopathy and emotion processing currently appears invariant. In addition, the relationship between psychopathic traits and reduced emotional responsiveness may be less robust than most contemporary theoretical perspectives suggest across a broad range of tasks. Our meta-analyses failed to provide evidence that either an overall measure of psychopathic traits or specific measures of the core affective and interpersonal traits of psychopathy are associated with reduced emotional reactivity. Given the scarcity of research data that allows such comparison, we urge researchers to use both types of measures.

## Supporting information

Eisenbarth et al. supplementary material Eisenbarth et al. supplementary material

## Supplementary material

The supplementary material for this article can be found at http://doi.org/10.1017/S109285292500015X.
