# Supplementary material for: Comparing the relationship between emotional responsiveness and psychopathy across assessment types: a systematic review and meta-analysis
Source: CNS Spectr. 2025 Mar 10;30(1):e32. doi: 10.1017/S109285292500015X (PMC13064751; doi:10.1017/S109285292500015X)
Supplement: Eisenbarth et al. supplementary material [file S109285292500015Xsup001.docx]

**Supplementary materials**

**A Figures**

**Figure S1**

*QQ-Plot for All Effect Sizes for Psychopathy Total Scores*


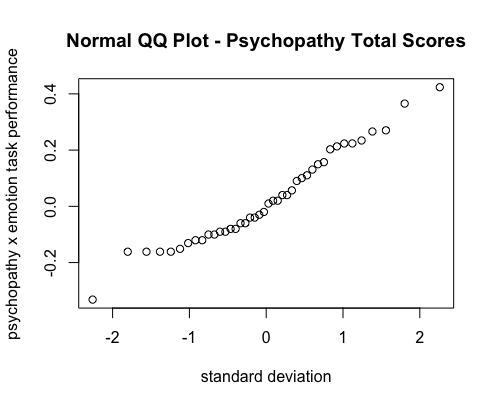


**Figure S2**

*Box Plot for all Effect Sizes for Psychopathy Total Scores*


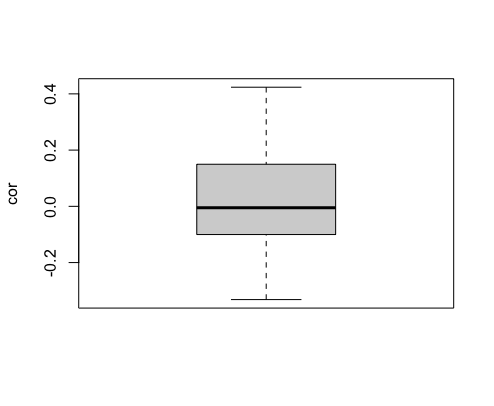


**B Meta-analyses with tasks that ensure clear predictions about emotional responsiveness**

The interpretation of effect sizes for risk taking and for Go/NoGo tasks may be viewed as ambiguous. In particular, on a risk-taking task, excessive anticipatory excitement about reward might lead to the same kind of performance as insufficient reactivity to punishment cues. Similarly, on Go/NoGo tasks, either heightened excitement about the possibility of reward or reduced concern about the possibility of punishment could explain a greater number of responses coded as false alarms. In principal analyses, where these tasks were included, we treated risk-taking and Go/NoGo tasks as measures of responsiveness to cues for punishment; consequently, higher scores on these tasks were coded as reflecting greater emotional responsiveness. However, because these measures do not provide unambiguous indices of responsiveness to emotion (i.e., they test whether people are more responsive to one contingency than to an opposite contingency), we conducted a second set of analyses that excluded these tasks.

**Descriptive statistics**

Excluding effect sizes from risk taking and Go/NoGo tasks resulted in 28 effect sizes. The studies included predominantly participants from offending populations (see Table 1), a mean sample size of 55.14 (*SD* = 39.87, range: 14-105) and had a mean effect size of 0.06 (*SD* = 0.18, range: -0.33 - 0.42).

**Meta-Analyses**

***Models Assessing Total Psychopathy Scores***

The level of heterogeneity was large, with approximately 29.93% of variation in effect size estimation attributable to variation among studies. The overall relationship between total psychopathy scores and emotional responsiveness was not significant (*k* = 28, pooled *r* = .01, *p* = .92, 95% CI[-.13,.14], *I^2^* = 29.93). This was true of the pooled relationship across both assessment types (*k* = 28, pooled *r* = -.02, *p* = .65, 95% CI[-.11,.08], *I^2^* = 31.94), and also when psychopathy was assessed specifically via clinical measures (pooled *r* = -.02, *p* = .59, 95% CI[-.13, .10]) or via self-report measures (pooled *r* = .06, *p* = .44, 95% CI[-.16,.28]). The heterogeneity in these separate analyses remained large for the self-report measures (*I^2^* = 54.45) but small for the PCL based pooled effect size (*I^2^* = 1.00). There was no statistical difference depending on type of psychopathy assessment *(t*(3) = 0.76, *p* = .50, CI[-.16,.26]).

***Models assessing Factor Scores***

**Factor 1.** The level of heterogeneity was moderate, with 33.68% of variation in effect size estimation attributable to variation among studies (Higgins et al., 2003). Overall, we identified no significant relationship between Factor 1 scores and scores on emotional responsiveness (*k* = 32, pooled *r* = .05, *p* = .37, 95% CI[-.09, .19]). Similarly, when broken down by assessment type, there was no reliable relationship between Factor 1 scores and emotional responsiveness when assessed via clinical measures (pooled *r* = .03, *p* = .53, 95% CI[-.12, .19], *I^2^* = 16.20) or when assessed via self-report measures (pooled *r* = .05, *p* = .46, 95% CI[-.15, .25], *I^2^* = 40.31). The magnitude of the relationship did not differ significantly as a function of the psychopathy assessment type (*t*(4) = -.61, *p* = .58, CI[-.15,.10]).

**Factor 2.** The level of heterogeneity was moderate, with 34.95% of variation in effect size estimation attributable to variation among studies (Higgins et al., 2003). We found no evidence of a relationship between Factor 2 scores and emotional responsiveness (*k* = 28, pooled *r* = -.03, *p* = .57, 95% CI[-.16, .10]). This was also found when the effects of psychopathic traits were compared separately for clinically-based (pooled *r* = - .03, *p* = .49, 95% CI[-.16, .09], *I^2^* = 36.17) or self-report-based (pooled *r* = -.01, *p* = .88, 95% CI[-.27, .25], *I^2^* = 10.02) measures. The magnitude of the relationship did not differ significantly as a function of the psychopathy assessment type (*t*(3) = 0.97*,* *p* = .27, CI[-.15,.29]).
